# End-of-life discussions reduce the utilization of life-sustaining treatments during the last three months of life in cancer patients

**DOI:** 10.1038/s41598-022-11586-x

**Published:** 2022-05-06

**Authors:** Shang-Yih Chan, Yun-Ju Lai, Yu-Yen Chen, Shuo-Ju Chiang, Yi-Fan Tsai, Li-Fei Hsu, Pei-Hung Chuang, Chu-Chieh Chen, Yung-Feng Yen

**Affiliations:** 1 Department of Internal Medicine, Yangming Branch, Taipei City Hospital, Taipei, Taiwan; 2 Department of Health Care Management, National Taipei University of Nursing and Health Sciences, Taipei, Taiwan; 3 University of Taipei, Taipei, Taiwan; 4 School of Medicine, National Yang Ming Chiao Tung University, Taipei, Taiwan; 5 Division of Endocrinology and Metabolism, Department of Internal Medicine, Puli Branch of Taichung Veterans General Hospital, Nantou, Taiwan; 6 Department of Exercise Health Science, National Taiwan University of Sport, Taichung, Taiwan; 7 Institute of Public Health, National Yang Ming Chiao Tung University, Taipei, Taiwan; 8 Department of Ophthalmology, Taichung Veterans General Hospital, Taichung, Taiwan; 9 School of Medicine, Chung Shan Medical University, Taichung, Taiwan; 10 Department of Cardiology, Yangming Branch, Taipei City Hospital, Taipei, Taiwan; 11 School of Biomedical Engineering, Taipei Medical University, Taipei, Taiwan; 12 Department of Nursing, Yangming Branch, Taipei City Hospital, Taipei, Taiwan; 13 School of Nursing, Taipei Medical University, Taipei, Taiwan; 14 Department of Allied Health Education and Digital Learning, National Taipei University of Nursing and Health Sciences, Taipei, Taiwan; 15 College of Public Health, National Taiwan University, Taipei, Taiwan; 16 Taipei Association of Health and Welfare Data Science, Taipei, Taiwan; 17 Section of Infectious Diseases, Yangming Branch Taipei City Hospital, Taipei City Government, No. 145, Zhengzhou Rd., Datong Dist., Taipei City, 10341 Taiwan; 18 Department of Education and Research, Taipei City Hospital, Taipei City, Taiwan

**Keywords:** Diseases, Health care

## Abstract

Studies to examine the impact of end-of-life (EOL) discussions on the utilization of life-sustaining treatments near death are limited and have inconsistent findings. This nationwide population-based cohort study determined the impact of EOL discussions on the utilization of life-sustaining treatments in the last three months of life in Taiwanese cancer patients. From 2012 to 2018, this cohort study included adult cancer patients, which were confirmed by pathohistological reports. Life-sustaining treatments during the last three months of life included cardiopulmonary resuscitation, intubation, and defibrillation. EOL discussions in cancer patients were confirmed by their medical records. Association of EOL discussions with utilization of life-sustaining treatments were assessed using multiple logistic regression. Of 381,207 patients, the mean age was 70.5 years and 19.4% of the subjects received life-sustaining treatments during the last three months of life. After adjusting for other covariates, those who underwent EOL discussions were less likely to receive life-sustaining treatments during the last three months of life compared to those who did not (Adjusted odds ratio [AOR] 0.87; 95% confidence interval [CI] 0.85–0.89). Considering the type of treatments, EOL discussions correlated with a lower likelihood of receiving cardiopulmonary resuscitation (AOR = 0.45, 95% CI 0.43–0.47), endotracheal intubation (AOR = 0.92, 95%CI 0.90–0.95), and defibrillation (AOR = 0.54, 95%CI 0.49–0.59). Since EOL discussions are associated with less aggressive care, our study supports the importance of providing these discussions to cancer patients during the EOL treatment.

## Introduction

Cancer is the second leading cause of death worldwide, and was responsible for an estimated 10 million deaths in 2020^1^. Patients often experience pain, dyspnea, and distress during the end-of-life (EOL) care. Aggressive EOL treatment has predicted lower quality of life^2,3^ and greater regret about treatment decisions^4^.

Advance care planning (ACP) is the process of discussing patient preferences regarding life-sustaining treatments and place of death during EOL care, and involves healthcare professionals, patients, and their family^5^. It is considered a means for helping patients die in their preferred treatment and is emphasised as an end-of-life care strategy^5^. A previous report showed that patients engaging in ACP have more stable EOL preferences compared to those who do not^6^.

Comprehensive EOL discussions with physicians during the ACP consultation could allow cancer patients to confront the limitations of medical treatments and consider their preferences regarding life-sustaining treatments through the EOL treatment. A meta-analysis review showed that these discussions predicted an increased use of hospice care and better quality of death^7^. Although cancer patients may avoid discussing their preference of life-sustaining treatments during EOL discussions^8,9^, healthcare providers are allowed—or morally obligated—to decide whether life-sustaining treatments are viable for terminal cancer patients^10^.

Although EOL discussions offer patients the opportunity to define their expectations for life-sustaining treatments that they want to receive during the EOL treatment, the impact of EOL discussions on the use of life-sustaining treatments near death has not been extensively studied. A US cohort study followed up 332 patients with advanced cancer and found that patients who participated in EOL discussions were less likely to receive ventilation (adjusted odds ratio [AOR] 0.26; 95% confidence interval [CI] 0.08–0.83) and resuscitation (AOR 0.16, 95% CI 0.03–0.80) during the EOL treatment compared to those who did not^8^. In another observational study in the US involving 145 patients with advanced cancer, EOL discussions lowered the rates of ventilation and resuscitations during the EOL treatment^2^. Previous studies on the association between EOL discussions and the use of life-sustaining treatment, however, have used small sample sizes (n = 145)^2^ and inadequately controlled for potential confounders such as dementia^2,8^.

EOL discussions serve to maximize patients’ quality of life while considering their goals and expectations regarding EOL treatment. It is important to understand the impact of these discussions on the administration of life-sustaining treatments among terminally ill patients. We therefore conducted a nationwide population-based cohort study to determine the impact of EOL discussions on the utilization of life-sustaining treatments during the last three months of life among Taiwanese cancer patients, from 2012 to 2018.

## Methods

### Promotion of end-of-life discussions in terminally ill patients in Taiwan

The Taiwan National Health Insurance Administration, since 2012, has initiated an EOL discussions program that encourages healthcare providers to provide these discussions to terminally ill patients^11^. While patients were admitted to the hospital, healthcare providers offered EOL discussions to those with life-limiting diseases^11^. During the discussions, healthcare providers discuss patients’ goals and preferences concerning medical care and life-sustaining treatments towards the end of their lives^11^. Each EOL discussion with a patient awards the healthcare provider with the equivalent of US$75^11^. There should be a record of all discussion content in the patient’s medical chart and each session must be at least an hour long^11^.

### Study population

This nationwide cohort study used the Taiwan National Health Insurance Research Database (NHIRD), which contains healthcare data from more than 99% of the population in Taiwan^12^. It is a large-scale database derived from the national health insurance system, which consists of registration files and original claims data. The database de-identified and scrambled the patients’ identification codes before releasing the data for research purposes^13^.

This cohort study selected subjects aged 18 years or older who had received a cancer diagnosis and died between January 1, 2012 and December 31, 2018. We identified these patients from the Registry for Catastrophic Illness. In Taiwan, the Registry for Catastrophic Illness requires a peer review of pathohistological reports before a cancer diagnosis is reported^14^. This registry was also linked to Taiwan’s death certificate database to confirm the demise of cancer patients^15^. The Institutional Review Board of Taipei City Hospital (no. TCHIRB-10709107-W) approved this study. The informed consents for study participants were waived by the Institutional Review Board of Taipei City Hospital. All methods in this study were performed in accordance with relevant guidelines and regulations. This study is also in accordance with the Declaration of Helsinki.

### Outcome variable

The outcome was life-sustaining treatments during the last three months of life in cancer patients. Life-sustaining treatments included cardiopulmonary resuscitation, intubation, and defibrillation^16^.

### Main explanatory variable

The main explanatory variable was EOL discussions with physicians, which was determined by patients’ medical records.

### Controlling variables

The controlling variables included sociodemographic characteristics, type of cancer, and comorbidities. Sociodemographic factors included age, sex, urbanization, and income level. Urbanization described whether subjects resided in urban, suburban, or rural areas^17^. We calculated the income level from the average monthly income of the insured person and grouped it into three categories: low (≤ 19,200 New Taiwan Dollars [NTD]), intermediate (19,201 NTD to < 40,000 NTD), and high (≥ 40,000 NTD). Type of cancer and the comorbidities in the study subjects were determined according to the International Classification of Diseases, Ninth and Tenth Revision, Clinical Modification (ICD-9-CM and ICD-10-CM) code. The type of cancer included solid tumors and hematologic malignancies. Subjects’ comorbidities include diabetes, chronic kidney disease, congestive heart failure, coronary heart disease, liver cirrhosis, chronic obstructive pulmonary disease, dementia, cerebrovascular disease, and depressive disorder (Supplementary Table S1). A person was considered to have a comorbidity only if the condition occurred in an inpatient setting or in three or more outpatient visits^18^.

### Statistical analysis

First, we analyzed the subjects’ demographic data. We then analyzed categorical data using the Pearson χ^2^ test where appropriate. We presented continuous data as mean ± standard deviation (SD), and conducted a two-sample t-test to compare outcomes between patients who underwent EOL discussions and those who did not.

We assessed the crude associations of EOL discussions and other covariates with the outcome (utilization of life-sustaining treatments during the last three months of life) by computing the odds ratios (ORs) and corresponding 95% confidence intervals (CIs). We then performed a multivariate logistic regression to estimate the association between EOL discussions and the utilization of life-sustaining treatments after adjusting for potential confounders. A variable with p < 0.05 was defined as a significant factor associated with the utilization of life-sustaining treatments in the multivariate analysis. Adjusted odds ratios (AOR) with 95% confidence intervals (CI) indicated the strength and direction of these associations.

We conducted subgroup and sensitivity analyses to examine the associations between EOL discussions and the utilization of life-sustaining treatments, after stratifying participants by age and sex. To examine the robustness of the main findings, propensity score analysis was conducted to evaluate the associations between hospice care services and utilization of life-sustaining treatments during the last three months of life. Logistic regression was conducted to calculate the probability of enrolling in a hospice care program in cancer patients according to their age, sex, income level, type of cancer, and comorbidities. A multivariate logistic regression was used to estimate the association between hospice care services and utilization of life-sustaining treatments after adjusting for patients’ propensity score. We performed all data management and analyses using the SAS® v9.4 statistical software package (SAS Institute, Cary, NC, USA).

## Results

### Participant selection

This cohort study involved the raw data of 383,128 deceased cancer patients from 1 January 2012 to 31 December 2018. After excluding those younger than 18 years (*n* = 850) and those with missing data (*n* = 1071), 381,207 patients were eligible for the purposes of the study. The overall mean (SD) age was 70.5 (14.3) years; 65.6% of the subjects were male; and 14.1% had EOL discussions with healthcare providers towards the end of their lives. The median duration from the EOL discussion to death for the 53,783 patients completing EOL discussions with healthcare providers was 34 days (interquartile range 16–79 days); of these patients, 7382 (13.7%) had such discussions after utilizing life-sustaining treatment.

### Characteristics of patients by end-of-life discussions

Table 1 shows the characteristics of cancer patients with and without EOL discussions before death. The patients who participated in EOL discussions tended to be younger and were more likely to be female. The proportion of patients completing EOL discussions with healthcare providers significantly increased from 3.67% in 2012–2014 to 17.54% in 2015–2016 and 24.79% in 2017–2018 (p < 0.001). During the follow-up period, 74,034 patients utilized life-sustaining treatments during their last three months of life, including 9,048 (16.82%) patients who had EOL discussions and 64,986 (19.85%) who did not have EOL discussions.

**Table 1:** Characteristics of deceased cancer patients by end-of-life discussions.

Characteristics	No. (%) of subjects^a^			*P* value
	Total N = 381207	Patients with EOL discussions, n = 53,783	Patients without EOL discussions, n = 327,424	
**Demographics**				
Age, years				
Mean ± SD	70.46 ± 14.26	69.19 ± 14.21	70.66 ± 14.26	< .001
18–64	131,034 (34.37)	20,302 (37.75)	110,732 (33.82)	< .001
≥ 65	250,173 (65.63)	33,481 (62.25)	216,692 (66.18)	
Sex				< .001
Female	147,147 (38.60)	21,226 (39.47)	125,921 (38.46)	
Male	234,060 (61.40)	32,557 (60.53)	201,503 (61.54)	
Income level				< .001
Low	64,059 (16.80)	8745 (16.26)	55,314 (16.89)	
Intermediate	195,861 (51.38)	26,823 (49.87)	169,038 (51.63)	
High	121,287 (31.82)	18,215 (33.87)	103,072 (31.48)	
Urbanization				< .001
Rural	44,575 (11.69)	5358 (9.96)	39,217 (11.98)	
Suburban	232,349 (60.95)	32,814 (61.01)	199,535 (60.94)	
Urban	104,283 (27.36)	15,611 (29.03)	88,672 (27.08)	
Year of enrollment				
2012–2014	154,680 (40.58)	5680 (10.56)	149,000 (45.51)	< .001
2015–2016	111,081 (29.14)	19,484 (36.23)	91,597 (27.98)	
2017–2018	115,446 (30.28)	28,619 (53.21)	86,827 (26.52)	
Type of cancer				
Solid tumor	362,507 (95.09)	51,152 (95.11)	311,355 (95.09)	0.875
Hematologic malignancies	18,700 (4.91)	2631 (4.89)	16,069 (4.91)	
Comorbidity				
Diabetes	135,886 (35.65)	19,087 (35.49)	116,799 (35.67)	0.411
Chronic kidney disease	87,396 (22.93)	11,282 (20.98)	76,114 (23.25)	< .001
Congestive heart failure	59,996 (15.74)	7786 (14.48)	52,210 (15.95)	< .001
Coronary heart disease	124,070 (32.55)	16,296 (30.30)	107,774 (32.92)	< .001
Liver cirrhosis	57,255 (15.02)	7697 (14.31)	49,558 (15.14)	< .001
COPD	118,345 (31.04)	15,677 (29.15)	102,668 (31.36)	< .001
Dementia	37,402 (9.81)	4790 (8.91)	32,612 (9.96)	< .001
Cerebrovascular disease	102,614 (26.92)	13,501 (25.10)	89,113 (27.22)	< .001
Depressive disorder	45,021 (11.81)	6330 (11.77)	38,691 (11.82)	0.753
Outcome				
Life-sustaining treatments during the last 3 months of life	74,034 (19.42)	9048 (16.82)	64,986 (19.85)	< .001
Endotracheal intubation	66,861	8483 (15.77)	58,378 (17.83)	< .001
Cardiopulmonary resuscitation	32,739	2269 (4.22)	30,470 (9.31)	< .001
Defibrillation	6493	514 (0.96)	5979 (1.83)	< .001

EOL, end-of-life; SD, standard deviation; COPD, chronic obstructive pulmonary disease.

^a^Unless stated otherwise.

Of the 53,783 patients completing EOL discussions with healthcare providers, the average total hospital admission expenses during the last three months of life were 468,740 and 195,830 NTD in those receiving and not receiving life-sustaining treatments during their last three months of life, respectively (p < 0.001).

### Association of end-of-life discussions with utilization of life-sustaining treatments

Table 2 shows the univariate and multivariate analyses of factors associated with life-sustaining treatments during the last three months of life among the cancer patients. After adjusting for the sociodemographic factors and co-morbidities, those who underwent EOL discussions were less likely to receive life-sustaining treatments during the last three months of life, compared to those who did not (AOR = 0.87, 95% CI 0.85–0.89).

**Table 2:** Univariate and multivariate analysis of factors associated with utilization of life-sustaining treatments during the last 3 months of life among deceased cancer patients.

Variables	Number of patients	Life-sustaining treatments^a^	Univariate		Multivariate analysis	
		n (%)	OR (95%CI)	P value	AOR (95%CI)	P value
**EOL discussions**						
No	327,424	64,986 (19.85)	1		1	
Yes	53,783	9048 (16.82)	0.82 (0.80–0.84)	< .001	0.87 (0.85–0.89)	< .001
**Demographics**						
Age, years						
18–64	131,034	25,318 (19.32)	1		1	
≥ 65	250,173	48,716 (19.47)	1.01 (0.99–1.03)	0.263	0.85 (0.84–0.87)	< .001
Sex						
Female	147,147	24,875 (16.90)	1		1	
Male	234,060	49,159 (21.00)	1.31 (1.29–1.33)	< .001	1.32 (1.30–1.34)	< .001
Income level						
Low	64,059	13,046 (20.37)	1		1	
Intermediate	195,861	37,507 (19.15)	0.93 (0.91–0.95)	< .001	0.97 (0.95–0.99)	0.015
High	121,287	23,481 (19.36)	0.94 (0.92–0.96)	< .001	0.99 (0.97–1.01)	0.395
Urbanization						
Rural	44,575	8754 (19.64)	1		1	
Suburban	232,349	45,238 (19.47)	0.99 (0.96–1.02)	0.407	0.99 (0.96–1.01)	0.301
Urban	104,283	20,042 (19.22)	0.97 (0.95–1.00)	0.06	0.97 (0.95–1.00)	0.082
Year of enrollment						
2012–2014	154,680	32,217 (20.83)	1		1	
2015–2016	111,081	21,204 (19.09)	0.90 (0.88–0.91)	< .001	0.90 (0.89–0.92)	< .001
2017–2018	115,446	20,613 (17.86)	0.83 (0.81–0.84)	< .001	0.84 (0.82–0.85)	< .001
Type of cancer						
Solid tumor	362,507	68,387 (18.87)	1			
Hematologic malignancies	18,700	5647 (30.20)	1.86 (1.80–1.92)	< .001	1.85 (1.79–1.91)	< .001
**Comorbidity**						
Diabetes						
No	245,321	45,400 (18.51)	1		1	
Yes	135,886	28,634 (21.07)	1.18 (1.16–1.20)	< .001	1.12 (1.10–1.14)	< .001
Chronic kidney disease						
No	293,811	54,038 (18.39)	1		1	
Yes	87,396	19,996 (22.88)	1.32 (1.29–1.34)	< .001	1.21 (1.18–1.23)	< .001
Congestive heart failure						
No	321,211	59,746 (18.60)	1		1	
Yes	59,996	14,288 (23.81)	1.37 (1.34–1.40)	< .001	1.24 (1.21–1.27)	< .001
Coronary heart disease						
No	257,137	46,786 (18.19)	1		1	
Yes	124,070	27,248 (21.96)	1.27 (1.24–1.29)	< .001	1.14 (1.12–1.16)	< .001
Liver cirrhosis						
No	323,952	63,979 (19.75)	1		1	
Yes	57,255	10,055 (17.56)	0.87 (0.85–0.89)	< .001	0.85 (0.83–0.87)	< .001
COPD						
No	262,862	49,016 (18.65)	1		1	
Yes	118,345	25,018 (21.14)	1.17 (1.15–1.19)	< .001	1.06 (1.04–1.08)	< .001
Dementia						
No	343,805	66,583 (19.37)	1		1	
Yes	37,402	7451 (19.92)	1.04 (1.01–1.06)	0.009	0.96 (0.93–0.98)	0.002
Cerebrovascular disease						
No	278,593	51,892 (18.63)	1		1	
Yes	102,614	22,142 (21.58)	1.20 (1.18–1.22)	< .001	1.11 (1.09–1.14)	< .001
Depressive disorder						
No	336,186	64,788 (19.27)	1			
Yes	45,021	9246 (20.54)	1.08 (1.06–1.11)	< .001	1.07 (1.04–1.09)	< .001

AOR, adjusted odds ratio; CI, confident interval; EOL, end-of-life; COPD, chronic obstructive pulmonary disease.

^a^During the last 3 months of life.

### Association of end-of-life discussions with cardiopulmonary resuscitation, endotracheal intubation, and defibrillation

Table 3 shows the multivariate analyses for the association of EOL discussions with cardiopulmonary resuscitation, endotracheal intubation, and defibrillation in cancer patients. After adjusting for the sociodemographic factors and co-morbidities, patients who underwent EOL discussions were less likely to receive cardiopulmonary resuscitation (AOR = 0.45, 95% CI 0.43–0.47), endotracheal intubation (AOR = 0.92, 95% CI 0.90–0.95), and defibrillation (AOR = 0.54, 95% CI 0.49–0.59) during their last three months of life, compared to those who did not.

**Table 3:** Multivariate analysis for the association of end-of-life discussions with cardiopulmonary resuscitation, endotracheal intubation, and defibrillation in deceased cancer patients.

Variables	Cardiopulmonary resuscitation^a^		Intubation^a^		Defibrillation^a^	
	AOR (95% CI)	P value	AOR (95% CI)	P value	AOR (95% CI)	P value
**EOL discussions**	0.45 (0.43–0.47)	< .001	0.92 (0.90–0.95)	< .001	0.54 (0.49–0.59)	< .001
**Age, years**						
18–64	1		1		1	
≥ 65	0.90 (0.88–0.93)	< .001	0.86 (0.84–0.88)	< .001	0.74 (0.70–0.79)	< .001
**Gender**						
Male	1.25 (1.22–1.28)	< .001	1.31 (1.29–1.34)	< .001	1.31 (1.24–1.38)	< .001
**Income level**						
Low	1		1		1	
Intermediate	0.93 (0.90–0.96)	< .001	0.99 (0.96–1.01)	0.262	1.10 (1.02–1.18)	0.010
High	0.91 (0.88–0.94)	< .001	1.00 (0.98–1.03)	0.982	1.17 (1.08–1.26)	< .001
**Urbanization**						
Rural	1		1		1	
Suburban	1.00 (0.97–1.04)	0.919	0.98 (0.96–1.01)	0.226	1.03 (0.95–1.11)	0.539
Urban	1.01 (0.97–1.06)	0.525	0.95 (0.93–0.98)	0.003	1.10 (1.00–1.20)	0.047
**Year of enrollment**						
2012–2014						
2015–2016	0.95 (0.93–0.98)	0.001	0.90 (0.88–0.92)	< .001	0.95 (0.89–1.01)	0.089
2017–2018	0.90 (0.88–0.93)	< .001	0.83 (0.82–0.85)	< .001	0.91 (0.85–0.96)	0.002
**Type of cancer**						
Solid tumor						
Hematologic malignancies	1.13 (1.07–1.19)	< .001	1.93 (1.87–2.00)	< .001	1.43 (1.30–1.57)	< .001
**Comorbidity**						
Diabetes	1.15 (1.12–1.17)	< .001	1.12 (1.10–1.14)	< .001	1.18 (1.12–1.24)	< .001
Chronic kidney disease	1.18 (1.15–1.21)	< .001	1.20 (1.18–1.23)	< .001	1.30 (1.23–1.38)	< .001
Congestive heart failure	1.35 (1.31–1.39)	< .001	1.22 (1.19–1.25)	< .001	1.96 (1.84–2.09)	< .001
Coronary heart disease	1.21 (1.18–1.25)	< .001	1.14 (1.12–1.16)	< .001	1.60 (1.51–1.70)	< .001
Liver cirrhosis	0.68 (0.66–0.71)	< .001	0.89 (0.86–0.91)	< .001	0.72 (0.66–0.78)	< .001
COPD	1.03 (1.00–1.06)	0.030	1.05 (1.03–1.07)	< .001	0.87 (0.82–0.92)	< .001
Dementia	1.04 (1.00–1.08)	0.060	0.93 (0.90–0.96)	< .001	0.81 (0.74–0.88)	< .001
Cerebrovascular disease	1.02 (1.00–1.05)	0.111	1.12 (1.10–1.15)	< .001	0.97 (0.92–1.03)	0.321
Depressive disorder	1.11 (1.07–1.15)	< .001	1.05 (1.03–1.08)	< .001	0.93 (0.86–1.00)	0.064

AOR, adjusted odds ratio; CI, confident interval; EOL, end-of-life; COPD, chronic obstructive pulmonary disease.

^a^During last 3 months of life.

### Subgroup and sensitivity analysis for the association between end-of-life discussions and life-sustaining treatments

Table 4 shows the results of subgroup analyses of the association between EOL discussions and life-sustaining treatments after stratifying participants by age and sex. In all patient subgroups, the discussions significantly predicted a lower likelihood of receiving life-sustaining treatments towards the end-of-life.

**Table 4:** Sensitivity analysis for the associations between end-of-life discussions and life-sustaining treatment after adjusting for patient characteristics.

Study subgroups	Life-sustaining treatments		Cardiopulmonary resuscitation		Endotracheal intubation		Defibrillation	
	AOR (95% CI)	P value	AOR (95% CI)	P value	AOR (95% CI)	P value	AOR (95% CI)	P value
All patients (n = 381,207)	0.87 (0.85–0.89)	< .001	0.45 (0.43–0.47)	< .001	0.92 (0.90–0.95)	< .001	0.54 (0.49–0.59)	< .001
Aged 18–64 (n = 131,034)	0.85 (0.81–0.88)	< .001	0.46 (0.42–0.49)	< .001	0.89 (0.86–0.93)	< .001	0.57 (0.49–0.66)	< .001
Aged ≥ 65 (n = 250,173)	0.89 (0.86–0.91)	< .001	0.44 (0.42–0.47)	< .001	0.94 (0.91–0.97)	< .001	0.53 (0.47–0.59)	< .001
Female patients (n = 147,147)	0.87 (0.84–0.91)	< .001	0.45 (0.42–0.48)	< .001	0.92 (0.88–0.96)	< .001	0.55 (0.47–0.64)	< .001
Male patients (n = 234,060)	0.87 (0.84–0.90)	< .001	0.45 (0.42–0.47)	< .001	0.92 (0.90–0.95)	< .001	0.54 (0.48–0.60)	< .001

AOR, adjusted odds ratio; CI, confident interval.

### Propensity score analysis for the association between end-of-life discussions and utilization of life-sustaining treatments

Propensity score analysis showed that cancer patients with EOL discussions were less likely to receive life-sustaining treatments during the last three months of life than those without the discussion (AOR = 0.87, 95% CI 0.85–0.89), including cardiopulmonary resuscitation (AOR = 0.45, 95% CI 0.43–0.47), endotracheal intubation (AOR = 0.92, 95% CI 0.90–0.94), and defibrillation (AOR = 0.54, 95% CI 0.49–0.59).

## Discussion

This nationwide cohort study found that 19.4% of cancer patients utilized life-sustaining treatments during the last three months of life. After adjusting for demographic data and comorbidities, cancer patients who underwent EOL discussions were less likely to receive life-sustaining treatments during EOL care. With regard to the treatment types, EOL discussions predicted a lower likelihood of receiving cardiopulmonary resuscitation, endotracheal intubation, and defibrillation towards the end of the patients’ lives.

Our study revealed robust associations between EOL discussions and life-sustaining treatments after stratifying patients by age and sex. In all patient subgroups, the discussions significantly lowered the likelihood of receiving life-sustaining treatments near death.

This study found that 14.1% of cancer patients in Taiwan participated in EOL discussions, which was lower than 37.0% among cancer patients in the US^8^. The culture difference regarding death-related issues between Asia and Western countries may explain the lower rate of completing EOL discussions among Taiwanese cancer patients. In traditional Chinese culture, death-related topics are thought to be taboo, and mentioning it is sacrilegious and to be avoided^19^. A previous survey in Hong Kong reported that 73% of elderly patients with chronic diseases have never received discussions regarding their EOL treatment^19^. Since EOL discussions provide patients the opportunity to define their goals and expectations regarding the EOL treatment^20^, our study suggests that it is imperative to provide such discussions for cancer patients nearing the end of their lives.

This study found that 19.4% of cancer patients in Taiwan received life-sustaining treatments in the last three months of life. A previous report showed that the proportion of cancer patients receiving intensive care in the last six months of life was 40.3% in the US, 17.5% in Belgium, 15.2% in Canada, and 8.2% in Germany^21^. Since intensive care in patients with terminal diseases is associated with lower quality of life^3^, our study suggests that healthcare professionals should proactively provide EOL discussions for terminally ill patients to discuss their preferred treatment during EOL care.

The present cohort study found that cancer patients who underwent EOL discussions had 18% lower rates of receiving life-sustaining treatments in the last three months of life, than those who did not. EOL discussions could allow terminally ill patients to consider their preferences regarding life-sustaining treatments during the EOL care. A previous report showed that EOL discussions significantly increased the rates of do-not-resuscitate order completion among advanced cancer patients^22^. Another cohort study in the US found that EOL discussions correlated with lower rates of ventilation and resuscitation near death^8^. Moreover, a study in the US analyzed 145 cancer patients and found that patients who had undergone EOL discussions had lower rates of receiving ventilation and resuscitations in the last week of life^2^. Our cohort study followed up 381,207 cancer patients and found that EOL discussions associated with a lower utilization of life-sustaining treatments during the last three months of life. With regard to treatment types, cancer patients who had EOL discussions had a lower likelihood of receiving cardiopulmonary resuscitation, endotracheal intubation, and defibrillation during EOL care. Thus the findings of our study suggest that EOL discussions could reduce the utilization of life-sustaining treatments in cancer patients near death.

The EOL conversations with physicians improve the patients’ understanding of the futility of aggressive therapies during EOL care^7^. This may account for the lower rate of receiving life-sustaining treatments at that stage. While cancer patients had EOL discussions with physicians, the benefit and harm of life-sustaining treatments during the EOL care would be discussed and emphasized. Through the EOL discussions with healthcare providers, cancer patients could consider and document their treatment preferences during EOL care. A prior study showed that EOL discussions significantly improved patients’ understanding of their terminal illness and subsequently reduced the likelihood of receiving aggressive treatments during EOL care^23^. As EOL discussions are associated with less aggressive care^7^, the findings of our study suggest that it is important to provide them for cancer patients during EOL treatment.

This study showed that cancer patients receiving life-sustaining treatments during EOL care had higher medical expenditures than those not. A previous study involving 314 veterans in the US showed that aggressive EOL treatment was associated with significantly higher inpatient costs during a terminal hospitalization^24^. Another US study using administrative data found that EOL discussion was associated with significant hospital cost savings among patients during EOL care^25^. Since aggressive EOL treatment is associated with higher health care cost and poor quality of EOL care in terminally ill patients^2^, our study suggests that EOL discussions should be provided for cancer patients to discuss their treatment preference during EOL care.

This nationwide cohort study has several strengths. First, this study is the largest cohort study to determine the impact of EOL discussions on the utilization of life-sustaining treatments during the last three months of life among cancer patients. The research design enhances the validity of our findings by including unbiased subject selection and strict cancer diagnostic criteria. Moreover, since the Taiwan National Health Insurance covers all medical care of cancer patients, this nationwide population-based study traced all cancer patients, thus minimizing referral bias. Furthermore, the study’s large sample size sufficiently detected the real, albeit subtle, difference between cancer patients who had and those who had not received EOL discussions towards the end of their lives.

This cohort study has several limitations. First, there may be important factors (e.g., patients’ religion, functional status, and educational level) associated with the decision of receiving life-sustaining treatments, which the National Health Insurance Research Database did not record. Second, of the 53,783 patients completing EOL discussions with healthcare providers, 7382 (13.7%) had such discussions after utilizing life-sustaining treatment, which would underestimate the impact of EOL discussions on the utilization of life-sustaining treatments during the last three months of life among cancer patients. Finally, since all our enrollees were Taiwanese, the external validity of our findings may be a concern. Therefore, the generalizability of our results to other non-Asian ethnic groups requires further verification. However, our findings suggest new avenues for future research.

In summary, this nationwide, cohort study found that cancer patients who had EOL discussions were less likely to receive life-sustaining treatments during the last three months of life compared to those who did not have EOL discussions. With regard to treatment types, EOL discussions correlated with a lower likelihood of receiving cardiopulmonary resuscitation, endotracheal intubation, and defibrillation during the last three months of life. As end-of-life discussions are associated with less aggressive EOL care, it is important to provide them for cancer patients during EOL treatment.

## Supplementary Information

Supplementary Information.
