# Supplementary material for: End-of-life discussions reduce the utilization of life-sustaining treatments during the last three months of life in cancer patients
Source: Sci Rep. 2022 May 6;12:7477. doi: 10.1038/s41598-022-11586-x (PMC9076633; doi:10.1038/s41598-022-11586-x)
Supplement: Supplementary file 1 — Supplementary Information. [file 41598_2022_11586_MOESM1_ESM.docx]

| Comorbidities | ICD-9-CM code | ICD-10-CM code |
| --- | --- | --- |
| Diabetes | 250 | E08~E13 |
| Chronic kidney disease | 585, 586 | N18 |
| Congestive heart failure | 428, 398.91, 402.01, 402.11, 402.91, 404.01, 404.03, 404.11, 404.13, 404.91, 404.93 | I50, I0981, I110, I130, I132 |
| Coronary heart disease | 410~414 | I20~I25 |
| Liver cirrhosis | 571, 571.2, 571.5, 571.6 | K703, K717, K746 |
| Chronic obstructive pulmonary disease | 491, 492, 518.1, 518.2, 770.2 | J41~J44 |
| Dementia | 290 | F01~F03 |
| Cerebrovascular disease | 430~437 | G46.3, G46.4, I60~I66, I69 |
| **Supplementary table 1.** International classification of diseases, ninth and tenth revision, clinical modification codes for comorbidities. ICD-9-CM, International Classification of Diseases, Ninth Revision, Clinical Modification; ICD-10-CM, International Classification of Diseases, Tenth Revision, Clinical Modification. | | |
